# Origin and mechanism of neuroblastoma

**DOI:** 10.18632/oncoscience.360

**Published:** 2017-09-21

**Authors:** Shoma Tsubota, Kenji Kadomatsu

**Affiliations:** Department of Biochemistry, Nagoya University Graduate School of Medicine, Nagoya 466-8550, Japan

**Keywords:** epigenome, neural crest, neuroblastoma, Schwann cell, sympathoadrenal lineage

Neuroblastoma is the most common extracranial solid tumor in childhood, and develops mainly in the adrenal medulla but also in the sympathetic ganglia. Some cases of neuroblastoma show spontaneous regression. Particularly, stage 4S, where S stands for “special”, exhibits spontaneous regression despite multifocal tumors. Another interesting feature is that, with few exceptions, driver gene mutations are rare in neuroblastoma, while copy number alterations are common, including *MYCN* amplification, 17q gain, and others, suggesting the involvement of epigenetic regulation [1]. These features suggest that neuroblastoma tumorigenesis is closely related to the development of the sympathoadrenal lineage. However, the underlying mechanisms, even the origin of neuroblastoma, remain largely obscure. To elucidate those mechanisms, three important papers have been recently published [2-4].

**Figure 1:**
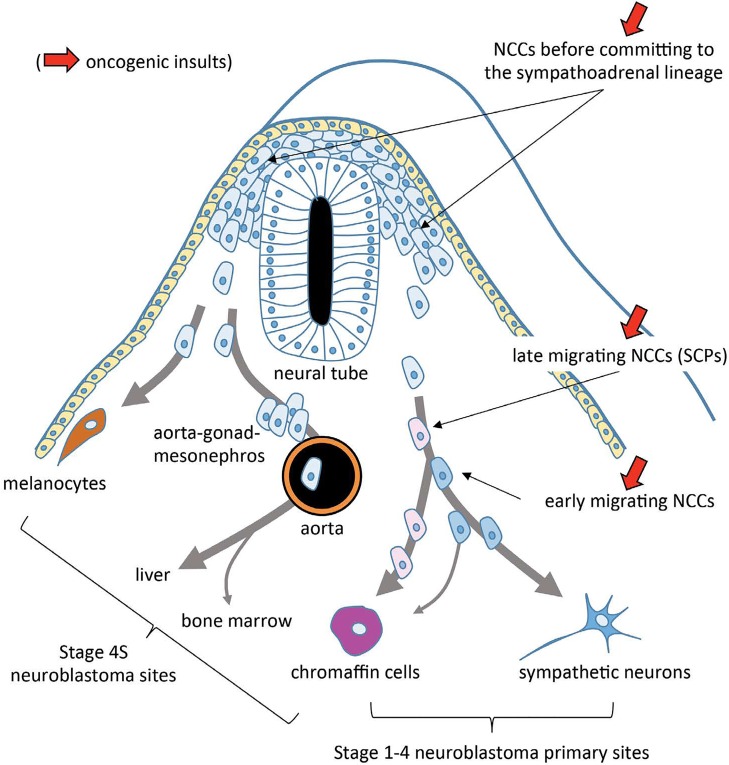
Origins of neuroblastoma

Neural crest cells (NCCs) are born at mid-gestation around the closure of the neural tube [embryonic day (E) 8.5-9.0 in mice]. Thereafter, NCCs migrate and give rise to a variety of tissues. In the sympathoadrenal lineage, NCCs reach their destinations close to the dorsal aorta at E9.5-10.0, where they proliferate, differentiate into neurons and glia, and finally form the sympathetic ganglia and adrenal medulla. Furlan et al. revisited this dogma [2]. Unexpectedly, they found that the majority of the adrenal medulla comes from peripheral stem cells called Schwann cell precursors (SCPs). Thus, early-migrating NCCs differentiate to sympathetic neurons at E10.5 and give rise to the suprarenal sympathetic ganglion and a small population of the adrenal medulla, while late-migrating NCCs differentiate into SCPs at E10.5 and migrate along with axons of preganglionic neurons in the neural tube that innervate the adrenal medulla. Importantly, SCPs are not only the source of Schwann cells, but also differentiate into chromaffin cells and dominate the chromaffin cell population in the adrenal medulla. Therefore, the sympathetic lineage and the adrenal lineage diverge unexpectedly early at E10.5 in mice. This finding suggest that there may be at least two possible origins of neuroblastoma: NCCs destined to become sympathetic neurons or chromaffin cells and SCPs destined to become chromaffin cells (Figure 1). The elucidated molecular profiles characteristic of SCPs will be useful for further studies on the mechanisms underlying neuroblastoma tumorigenesis.

Our group addressed the long-standing question of why neuroblastoma develops without driver gene mutations [3]. Using the neuroblastoma model TH-*MYCN* mice, we developed a spheroid culture method that well represents the in vivo status of neuroblasts at the molecular level. Passageable spheres could be established from sympathetic ganglia of E13.5 TH-*MYCN* mice, but not wild-type mice. Surprisingly, spheres from E13.5 TH-*MYCN* mice formed allograft tumors, suggesting that early molecular events of tumorigenesis occur no later than E13.5. Copy number alterations were late events, as they were found in advanced tumors, but not in E13.5 spheres, of TH-*MYCN* mice. Importantly, E13.5 spheres from TH-*MYCN* mice exhibited upregulation of MYC target genes and downregulation of polycomb repressive complex 2 (PRC2) target genes. Consistent with this, the H3K27me3 level was increased around transcription start sites of PRC2 targets in E13.5 spheres from TH-*MYCN* mice. Knockdown or inhibition of Ezh2, a methyltransferase in PRC2 catalyzing H3K27me3, led to a striking decrease in sphere formation and tumor formation in TH-*MYCN* mice. Moreover, expression of PRC2 targets reversely correlated with that of MYC targets in human neuroblastoma. Consistently, lower and higher expression of PRC2 targets and MYC targets, respectively, predicted a poor prognosis. Our study highlighted the importance of epigenetic regulation of *MYCN*-driven neuroblastoma, and revealed that tumorigenesis is initiated in unexpectedly early embryonic days (no later than E13.5) in this mouse model.

Olsen et al. provided another piece of evidence that NCCs could be a target of transformation [4]. Using E9.5 trunk neural tube, they enriched NCCs and expressed *MYCN*. *MYCN*-expressed NCCs developed subcutaneous tumors that exhibited chromosomal alterations and gene expression similar to those of *MYCN*-amplified human neuroblastoma. We also demonstrated that *MYCN* could transform spheres from E13.5 wild-type mice to passageable spheres [3]. This is surprising, considering that a combination of several oncogenes and suppressor genes is necessary to fully transform normal adult human cells, and that MYC or *MYCN* alone often induces apoptosis rather than transformation. These results collectively suggest that embryonic cells at mid-gestation, such as NCCs and SCPs, have a competency to be transformed if an appropriate insult, such as epigenetic dysregulation, occurs. In this context, a hypothesis proposed by van Noesel for the origin of stage 4S neuroblastoma is interesting [5]. Stage 4S bares multifocal tumors in the adrenal gland, liver, and skin as well as a small population in the bone marrow. It is known that NCCs migrate to the transient embryonic tissue called the “aorta-gonad-mesonephros”, through which they further move into the blood stream, liver, and bone marrow [6]. NCCs are observable in the liver at E14.5 and as a small population in the bone marrow at E18.5. Therefore, it is conceivable that initial events may occur in NCCs before they are committed to the sympathoadrenal lineage (Figure 1). Thus, neuroblastoma stage 4S could be a multifocal stem cell disease of the developing neural crest, not a consequence of multiple metastasis from a single clonal tumor [5].

The idea that neuroblastoma is a developmental disease of the neural crest will always be useful when we conduct further studies on this disease.
